# Draft Genome Sequence of Limosilactobacillus fermentum Strain NKN-51, Isolated from Fermented Yak Milk in the Western Himalayas of India

**DOI:** 10.1128/MRA.00186-21

**Published:** 2021-04-22

**Authors:** Arun Beniwal, Amit Gaurav, Rekha Sharma, Tamoghna Ghosh, Piyush Kumar, Urmila Netter, Ranjana Pathania, Naveen Kumar Navani

**Affiliations:** a Department of Biotechnology, Indian Institute of Technology Roorkee, Roorkee, Uttarakhand, India; Portland State University

## Abstract

Here, we report the draft genome sequence of Limosilactobacillus fermentum strain NKN-51, which was isolated from naturally processed yak cheese from the western Himalayas of India. The genome was assembled in 101 contigs with a total length of 1,879,705 bp and a GC content of 53.5%. Genome annotation predicted 1,730 protein-coding genes and 50 tRNA genes.

## ANNOUNCEMENT

Limosilactobacillus fermentum plays an important role in the production of fermented foods and has been reported to have human health benefits. Hence, Limosilactobacillus fermentum is recognized as a potential probiotic strain with attributes such as antimicrobial, antioxidative, and cholesterol reduction properties (1–4). The Himalayan region is home to diverse climatic conditions ranging from frozen glaciers to boiling springs. We isolated an *L. fermentum* strain from fermented butter made from yak milk (locally known as churpi) from Nubra Valley (34.6863°N, 77.5673°E) in the western Himalayas. Here, we report the genome sequence of *L. fermentum* strain NKN-51. It was isolated on de Man-Rogosa-Sharpe (MRS) agar and cultured in MRS broth (5). It was identified as *L. fermentum* with a microbial identification system (MicroStation; Biolog, USA) followed by 16S rRNA gene sequencing (6). In addition to the probiotic attributes of this strain, it produces the novel protein tyrosine phosphatase-like phytase (PTPLP) with high specificity for phytate only. We explored the technological potential of this phytase in an effort to understand the mechanistic details of phytase and found that it ameliorated the nutritional value of cereals and animal feed (6). The strain NKN-51 was subjected to genome sequencing for further studies and applications.

The total genomic DNA (gDNA) was extracted from a single isolated colony grown in MRS broth (HiMedia, India) using a PowerSoil DNA isolation kit (Qiagen, USA). The gDNA concentration was determined with the Qubit 3.0 fluorometer using the Qubit double-stranded DNA (dSDNA) high-sensitivity (HS) assay kit (Thermo Fisher Scientific, USA). A sequence library was constructed using the NEBNext Ultra II DNA library prep kit for Illumina (New England Biolabs, USA). Both quantity and quality checks of the amplified library were performed in a Bioanalyzer 2100 instrument (Agilent Technologies) using a high-sensitivity DNA chip per the manufacturer’s instructions. The Illumina HiSeq 4000 platform was used for sequencing the paired-end library, with a read length of 2 × 150 bp. Default parameters were used for all software unless otherwise specified. Processing of FASTQ sequence files for quality trimming was performed using Trim Galore! version 0.6.5 (https://www.bioinformatics.babraham.ac.uk/projects/trim_galore), and the quality assessment was performed with FastQC version 0.11.2 and MultiQC version 1.9 (7). *De novo* assembly of the bacterial genome was performed using Unicycler version 0.4.8 (8). The genome was assembled in 101 contigs, with a total length of 1,879,705 bp, an *N*_50_ value of 26,979 bp, a coverage depth of 545×, and a GC content of 53.5%. Draft genome annotation was performed using the NCBI Prokaryotic Genome Annotation Pipeline (9), which identified 1,862 coding sequences, 3 noncoding RNAs (ncRNAs), 50 tRNAs, 3 rRNA genes, and 132 pseudogenes. Analysis of secondary metabolite biosynthesis gene clusters using BAGEL4 revealed one cluster encoding potential bacteriocin (enterolysin_A) (10).

### Data availability.

The draft genome sequence of Limosilactobacillus fermentum NKN-51 was deposited in the NCBI database under the BioProject accession number PRJNA701539, BioSample accession number SAMN17885229, and SRA accession number SRR13711020.
